# Endovascular Repair of Thoracoabdominal Aortic Aneurysm under Locoregional Anesthesia

**DOI:** 10.3400/avd.cr.25-00152

**Published:** 2026-03-03

**Authors:** Kenichi Kato, Tsuyoshi Shibata, Yutaka Iba, Tomohiro Nakajima, Junji Nakazawa, Ayaka Arihara, Shigeki Komatsu, Masato Yonemori

**Affiliations:** Division of Cardiovascular Surgery, Department of Surgery, Sapporo Medical University, Sapporo, Hokkaido, Japan

**Keywords:** locoregional anesthesia, physician-modified inner-branched endovascular aneurysm repair, thoracoabdominal aortic aneurysm

## Abstract

An 80-year-old female presented with a thoracoabdominal aortic aneurysm (TAAA) that had progressively enlarged to a diameter of 58 mm. She was scheduled for TAAA repair; however, she had a severe obstructive ventilatory disorder, which posed significant risks. Both open repair and general anesthesia were deemed to carry a high risk of respiratory complications. Consequently, an endovascular TAAA repair was performed using a physician-modified inner-branched endograft under locoregional anesthesia. This approach successfully treated the TAAA without any major complications. This strategy opens up the possibility of treating TAAA in patients with severe comorbidities that were previously challenging to treat.

## Introduction

Open thoracoabdominal aortic aneurysm (TAAA) repair is a highly invasive radical surgical treatment. The incidence of mortality, morbidity, and postoperative respiratory failure is higher in patients with chronic obstructive pulmonary disease (COPD),^1)^ which makes it difficult to treat TAAA. Currently, fenestrated/branched endovascular repair is a treatment option for high-risk TAAA cases. Despite its use in older patients with higher rates of comorbidities, it has demonstrated outcomes comparable to those of open repair.^2)^ Although off-the-shelf or custom-made endografts for TAAA are still not available in Japan, we have performed physician-modified inner-branched endovascular aneurysm repair (PMiBEVAR) for high-risk TAAA patients.^3)^ Moreover, using locoregional anesthesia (LRA) instead of general anesthesia (GA) can reduce the incidence of postoperative respiratory complications.^4)^ We present a case of TAAA repair with PMiBEVAR under LRA in a patient with severe COPD-related respiratory disorder.

## Case Report

The modification technique for performing stent-grafts was approved by the Institutional Review Board of our university (study approval number: 20-005; date of approval: December 22, 2020).

**Fig. 1:**
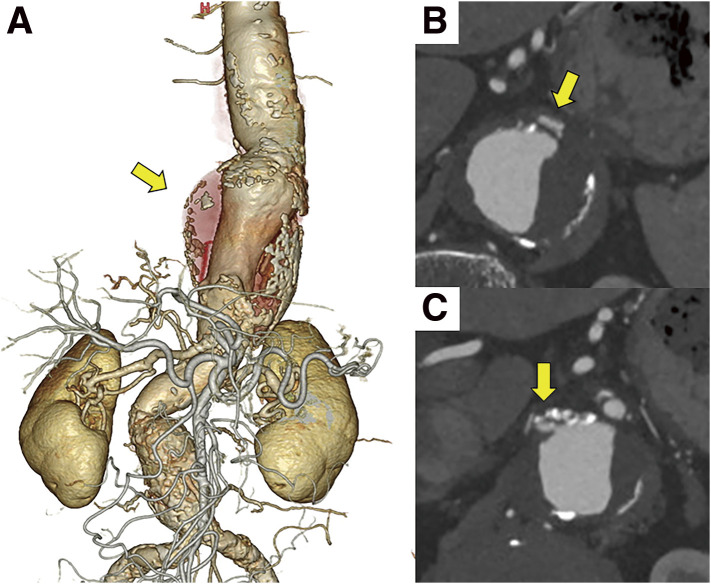
Preoperative contrast-enhanced CT. (**A**) Thoracoabdominal aneurysm (yellow arrow) in 3D CT angiography. (**B**) Celiac artery stenosis (yellow arrow). (**C**) Right renal artery stenosis (yellow arrow). CT: computed tomography

The patient was an 80-year-old female with a history of conservative treatment for acute type-B aortic dissection 10 years prior. Although the false lumen had fully regressed, a true TAAA developed and gradually dilated. The patient had COPD and a 30-year history of smoking half a pack per day. Contrast-enhanced computed tomography revealed a TAAA (maximum diameter of 58 mm) and stenosis of the celiac artery (CA) and right renal artery (RA) (**Fig. 1**). The Adamkiewicz artery (AKA) originated from the left first lumbar artery, and the CA communicated with the superior mesenteric artery (SMA) via the pancreaticoduodenal arcade. Spirometry showed severe obstructive ventilatory disorder (forced expiratory volume in 1 s [FEV1] of 0.56 L, FEV1/forced vital capacity of 34.4%, and FEV1/predicted FEV1 of 36.4%). Although surgical repair was planned for the enlarging TAAA, both open repair and GA were deemed high risk given the patient’s severe respiratory disorder. Spinal cord ischemia was also a concern because of the long treatment range and the need to sacrifice the AKA. To avoid these complications, a staged endovascular approach using PMiBEVAR under LRA (local anesthesia + ilioinguinal/iliohypogastric nerve block + genitofemoral nerve block) with conscious sedation was planned.

**Fig. 2:**
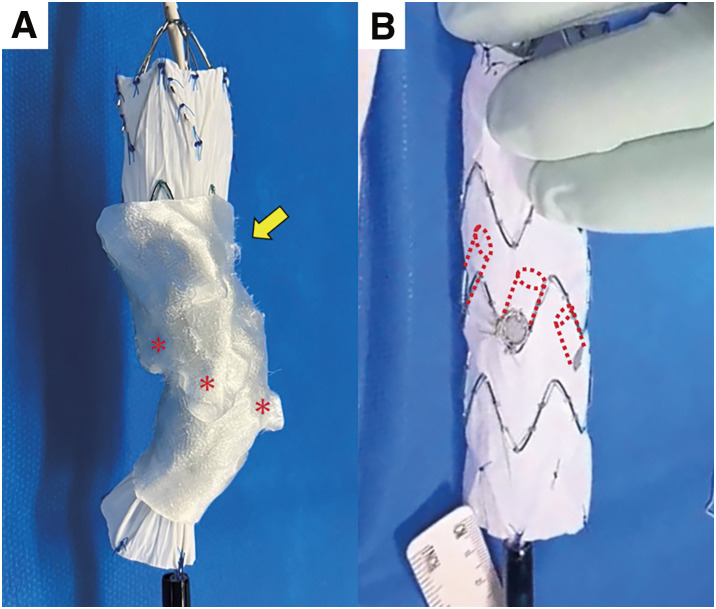
Device modification. (**A**) Device deployment in the 3D-printed aortic model (yellow arrow). Locations of the SMA and bilateral RAs (red asterisks) were marked and fenestrated. (**B**) Inner-branched endograft. The red dashed lines indicate the attached inner branches.

**Fig. 3:**
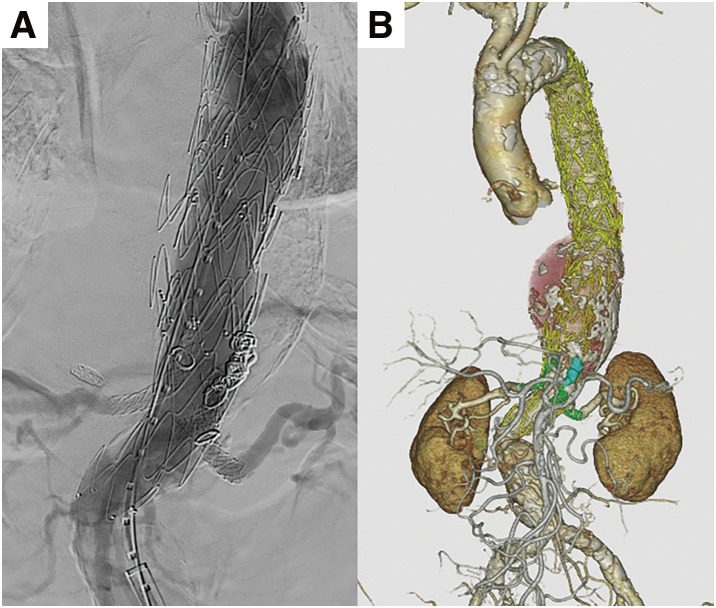
Angiography following device implantation. (**A**) Final digital subtraction angiography during the second surgery. (**B**) 3D-CT angiography after the second surgery.

During the first surgery, the CA was embolized with coils due to stenosis that made reconstruction difficult. A Relay Pro (Terumo, Tokyo, Japan) was deployed from the distal aortic arch to the CA level. The patient was discharged on postoperative day 10. The second surgery (PMiBEVAR) was performed on postoperative day 21. For device modification, a Zenith Alpha (Cook Medical, Bloomington, IN, USA) was deployed in the 3D-printed aortic model (**Fig. 2A**). The positions of the SMA and bilateral RAs were marked and fenestrated. Segments of Viabahn (W. L. Gore & Associates, Flagstaff, AZ, USA), cut into 7-mm pieces, were inserted into each fenestration and sewn as inner branches (**Fig. 2B**). The modified device was reloaded into the delivery system. The preoperative device modification time was 118 min (not included in the operation time). The details of the device modification are described in a previous article.^3)^ After balloon angioplasty to the right RA, the inner-branched device was deployed, aligning fenestrations with the abdominal branches. The SMA and bilateral RAs were reconstructed using bridge stents (Viabahn VBX; W. L. Gore & Associates). An additional endograft (Zenith Alpha) was deployed from the thoracic endograft to the inner-branched device. Intraoperative digital subtraction angiography and postoperative contrast-enhanced computed tomography showed no endoleaks and all branches were patent (**Fig. 3**). The operation time was 346 min, and the cumulative air kerma was 4669 mGy during the PMiBEVAR. Cannulation of the stenotic right RA took time. During the procedure, we confirmed that the patient could move both lower extremities in response to commands. Subsequently, the patient was discharged 10 days after PMiBEVAR without any respiratory or neurological complications.

Both surgeries were performed with percutaneous bilateral common femoral artery (CFA) puncture under LRA. The left CFA served as the main access site, and hemostasis was achieved using double Perclose ProStyle sutures (Abbott Vascular, Santa Clara, CA, USA) deployed before inserting the delivery system. Bridge stents were delivered from the left brachial artery. All procedures were performed under monitored anesthesia care provided by anesthesiologists. Sedation was achieved with a continuous infusion of dexmedetomidine (0.1–0.7 μg/kg/h) combined with bolus administrations of propofol (5–10 mg per time), and intravenous analgesia was provided with bolus doses of fentanyl (0.025–0.05 mg per time). Supplemental oxygen was administered via nasal cannula at 1 L/min during the first procedure and via a face mask at 1 L/min supplemented with 2 L/min of room air during the second procedure. Respiratory status was monitored using pulse oximetry and arterial blood gas analysis obtained from a radial arterial line. The procedures were completed under LRA without complaints of discomfort or notable patient movement.

## Discussion

This TAAA case was successfully repaired completely under LRA. A meta-analysis demonstrated that LRA reduced the rates of postoperative pneumonia and myocardial infarction as well as the hospital stay length compared with those for GA.^5)^ Additionally, in patients with COPD, LRA reduces the risks of postoperative pneumonia, prolonged ventilation, and unplanned intubation compared to GA.^4)^ Branched endografts have enabled TAAA repair under LRA, thereby facilitating treatment of patients with TAAA that was more challenging because of severe comorbidities.

Currently, several off-the-shelf branched endografts for TAAA repair are available, including the Zenith t-Branch (Cook Medical), Gore Excluder Thoracoabdominal Branch Endoprosthesis (W. L. Gore & Associates), and E-nside (Artivion, Kennesaw, GA, USA). However, a previous study reported that these devices are anatomically feasible in only 58% of TAAA patients.^6)^ Cook Medical provides custom-made devices that address anatomical limitations, but they require 6–12 weeks from planning to implantation; furthermore, risks of aneurysm rupture and target vessel occlusion during the lead period have been reported.^7)^ Severe stenosis of the target vessel, as seen in the right RA in this case, also increases the risk of occlusion during the waiting period.^7)^ Physician-modified devices can address both anatomical limitations and the risk of adverse events during this lead period. Another challenge with off-the-shelf and custom-made devices is limited availability. They are approved in only certain countries and remain unavailable in others, including Japan. Physician-modified devices enable endovascular TAAA repair even in such regions. The incidence of primary target vessel endoleaks is also lower for branched endografts than for fenestrated endografts.^8)^ An inner-branched design is considered advantageous for preventing endoleaks.

To protect the spinal cord, staged surgery was performed, which reduces the risk of postoperative spinal cord ischemia in endovascular TAAA repair compared to single-stage surgery.^9)^ LRA further supports spinal cord protection by avoiding GA-related intraoperative hypotension and allowing awake neurological monitoring for timely intervention.^10)^ Staged endovascular TAAA repair under LRA may be beneficial in high-risk patients.

In the present case, prolonged cannulation of the stenotic right RA led to an increased radiation dose. It did not exceed 5000 mGy, the level at which follow-up for radiation-induced skin injury is recommended,^11)^ and it may be considered acceptable when compared with the invasiveness of open TAAA repair. However, the median intraoperative radiation dose during elective endovascular TAAA repair using physician-modified endografts has been reported as 2002 mGy (interquartile range, 1083–3516 mGy),^12)^ and the dose in this case exceeded typical levels. One possible strategy to facilitate cannulation of the stenotic right RA would have been to place a bare metal stent during the first surgery. However, deploying a bridge stent within the bare metal stent could have resulted in a slight reduction of the luminal diameter due to the added stent thickness; and there was also concern that the guidewire might get caught on the stent during cannulation, making the procedure more challenging. For these reasons, we elected to perform balloon angioplasty to the right RA instead. In addition, performing balloon angioplasty during the first surgery raised the possibility of restenosis before the subsequent PMiBEVAR; therefore, we planned to perform the dilation at the time of PMiBEVAR. Although we anticipated that balloon angioplasty would facilitate reconstruction of the right RA, the procedure ultimately proved difficult. Furthermore, we have adopted the use of a 3D-printed aortic model to achieve precise positioning of the inner branches and thereby simplify branch reconstruction; however, several challenges remain with the branch reconstruction strategy.

Despite its advantages, LRA is generally considered less comfortable than GA. Conscious sedation is useful for alleviating discomfort, but patients may have difficulty holding their breath during digital subtraction angiography. Patients can also move during the procedure. Whether or not endovascular TAAA repair should be routinely performed under LRA is controversial. It might not be suitable when precise positioning is required. In this case, although the procedures were performed under LRA, intraoperative management was entrusted to anesthesiologists, allowing prompt and appropriate adjustments of sedation and analgesia in response to the patient’s condition and complaints. Through close collaboration with the anesthesiology team, the procedure was completed safely without notable discomfort or significant patient movement.

## Conclusion

TAAA accompanied by a severe respiratory disorder can be successfully repaired using PMiBEVAR under LRA without notable complications. This approach may expand treatment options for TAAA patients with severe comorbidities.
